# Unusual presentation of Pott disease and diagnostic challenges in a resource-limited setting: a case report

**DOI:** 10.1186/s13256-023-04015-8

**Published:** 2023-07-02

**Authors:** Anacret Byamukama, Moses Acan, Mugisha Julius Sebikali

**Affiliations:** Mbarara University of Science and Technology, Mbarara, Uganda

**Keywords:** Pott disease, Extrapulmonary tuberculosis, Paravertebral abscess, TB spondylodiscitis, Case report

## Abstract

**Background:**

Pott disease is rare and responsible for only 1%–2% of all tuberculosis cases. It poses diagnostic challenges in resource-limited settings due to unusual presentation and limited investigative capacity, resulting in debilitating sequelae if diagnosed late.

**Case presentation:**

We present a case of severe Pott disease of the lumbar spine, with a large paravertebral abscess tracking down to the gluteal region in a 27-year-old Black African Ugandan woman living with human immunodeficiency virus, whose main complaint was right lower abdominal pain. She was initially misdiagnosed from the peripheral clinics as a case of lumbago and later with a psoas abscess. The diagnosis of severe Pott disease was established at the regional referral hospital following an abdominal computed tomography scan, and the patient was appropriately initiated on anti-tuberculosis drugs. However, only abscess drainage and provision of a lumbar corset were possible, with no neurosurgical intervention done on the spine due to financial constraints. Clinical review at 2, 6, and 12 months revealed improvement.

**Conclusions:**

Pott disease may present with non-specific symptoms such as abdominal pain resulting from pressure effects of an expansile cold abscess. This, coupled with limited diagnostic capacity in resource-limited settings; results in significant morbidity and possible mortality. Hence, there is need to train clinicians to increase their index of suspicion and equip health units with basic radiological equipment, such as x-ray, for timely detection and subsequent management of Pott disease.

## Background

Pott disease, although rare, is a severe and often debilitating form of extrapulmonary tuberculosis, preferentially affecting and destroying vertebrae and intervertebral discs with permanent neurological sequelae if not diagnosed and managed early [1]. It is responsible for about 1%–2% of all tuberculosis (TB) cases and 50% of all musculoskeletal forms of TB, with the thoracolumbar vertebrae as the most affected [2]. People living with human immunodeficiency virus (HIV) have a five-fold risk of developing tuberculosis [3] and despite the wide availability of antiretroviral therapy (ART), tuberculosis (TB) is still a major health challenge in sub-Saharan Africa, and remains the single most leading cause of death among people living with HIV [4].

Because of its non-specific presentation, imaging is usually needed to arrive at a diagnosis of Pott disease [1], and yet not easily accessible in resource-limited settings, hence posing a diagnostic challenge [5].

Due to its debilitating effects if diagnosed late, it is crucial that health workers have a high index of suspicion for Pott disease and promptly initiate treatment.

Our case report aims to emphasize the fact that Pott disease can present with non-specific signs and symptoms that may not be suggestive of TB and highlights the diagnostic challenges in resource-limited settings.

## Case presentation

A 27-year-old Black African Ugandan woman living with HIV, on antiretroviral drugs for 10 years, was referred to Mbarara Regional Referral Hospital (MRRH) with a 2-month history of worsening right lower abdominal pain and low grade fevers. She had no history of cough, weight loss, or night sweats and had normal bowel and micturition habits.

Prior to her referral, she had been managed at a peripheral clinic as a case of a psoas abscess, and 2 years earlier at another peripheral clinic as a case of lumbago when she presented with mild lower back pain. She had been given supportive treatment that included amitriptyline and paracetamol (doses not specified), in addition to self-medication with local herbs. She reported to have initially experienced relief on the local and supportive medication, only to later develop lower abdominal pain that worsened with time. No diagnostic imaging had been carried out during that time.

She had been on tenofovir/lamivudine/efavirenz (TLE) (300 mg/300 mg/600 mg) and septrin (960 mg), then later transitioned to tenofovir/lamivudine/dolutegravir (TLD) (300 mg/300 mg/50 mg) in 2020, as per Uganda’s HIV treatment guidelines, and completed 6 months of TB preventive therapy (isoniazid for 6 months) in 2019. She had good ART adherence with suppressed viral loads (VLs) for the last two consecutive years. The most recent VL was 95 copies/mL (June 2021).

An abdominal ultrasound scan done at a peripheral clinic revealed an echo-complex, predominantly cystic mass of ~ 790 cc in the right lumbar region that was suspected to be a psoas abscess. This led to initial management of the patient as a case of a psoas abscess.

She had no other known chronic illness such as diabetes mellitus, hypertension, heart disease, or renal disease.

She had no history of trauma, blood transfusion, or surgical procedures.

She was a mother of one child, born by spontaneous vaginal delivery and had no history of pregnancy losses.

She was separated from the husband, a tailor by profession, and lived with the mother. There were no known familial illnesses.

No history of cigarette smoking or alcohol intake.

## Examination findings

On examination she was weak, of fair nutritional status, with no jaundice, no pallor of mucous membranes, and not dehydrated. Her blood pressure was 138/74 mmHg (millimeters of mercury), pulse—81 beats per minute (bpm), axillary temperature—37.2 °C, and oxygen saturation (SpO_2_)—99% at room air.

Per abdominal examination: Normal abdominal fullness, movement with respiration, and mild tenderness with a palpable mobile mass in the right iliac fossa. Bowel sounds were present and normal.

Neurological and musculoskeletal exam: The patient was fully conscious and oriented, GCS (Glasgow coma scale) was 15/15, pupils were equal and reactive to light (PEARL), with normal cranial nerve exam. Sensation and muscle power in both upper and lower limbs were intact. There was no neck stiffness and Kernig’s sign was negative. There was palpable tenderness and a gibbus in the lumbar spine region around L2/3 level.

Cardiovascular and respiratory exams were unremarkable.

## Laboratory findings

She had a normal full hemogram and serum electrolytes (Table 1). Liver function tests were not done.

**Table 1:** Chemistry and complete blood count test results

Test	Results
Chemistry	
Potassium Sodium Chloride	5.15 mmol/L (3.5–5.5) 139.8 mmol/L (135–155) 99.9 mmol/L (97–108)
Complete blood count	
White blood cell count (WBC) Hemoglobin (Hb) Platelet count	6.23 × 10^9^/L (3.5–9.5) 14.1 g/dL (11.5–15.0) 332 × 10^9^/L (125–350)

**Fig. 1:**
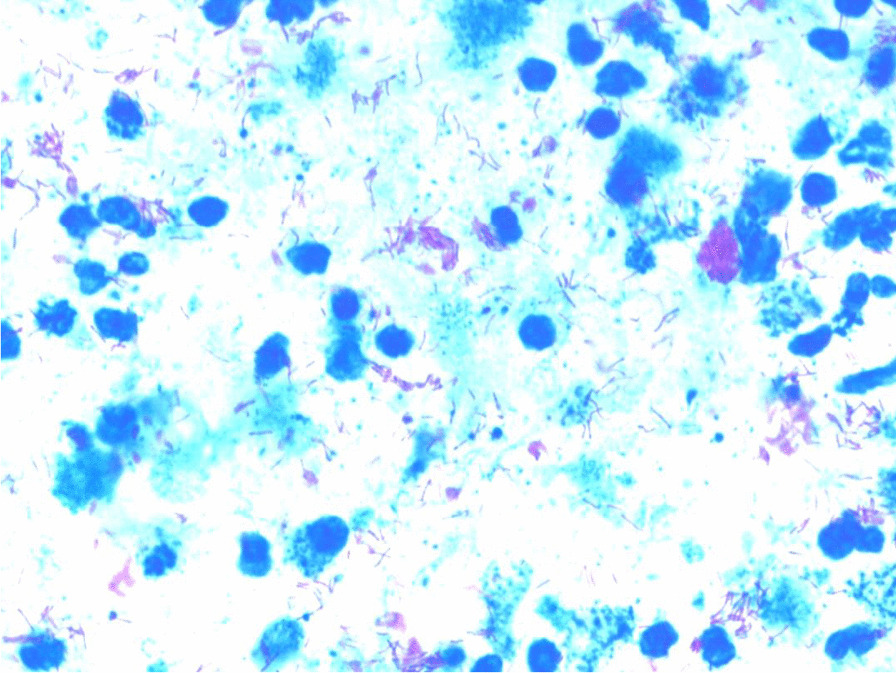
Acid-fast bacilli within the sample aspirate

A sample from the abscess showed acid-fast bacilli (AFB) (Fig. 1) on Ziehl–Neelson staining (ZN) and rifampicin sensitive *Mycobacterium tuberculosis* on gene-xpert.

## Imaging

**Fig. 2:**
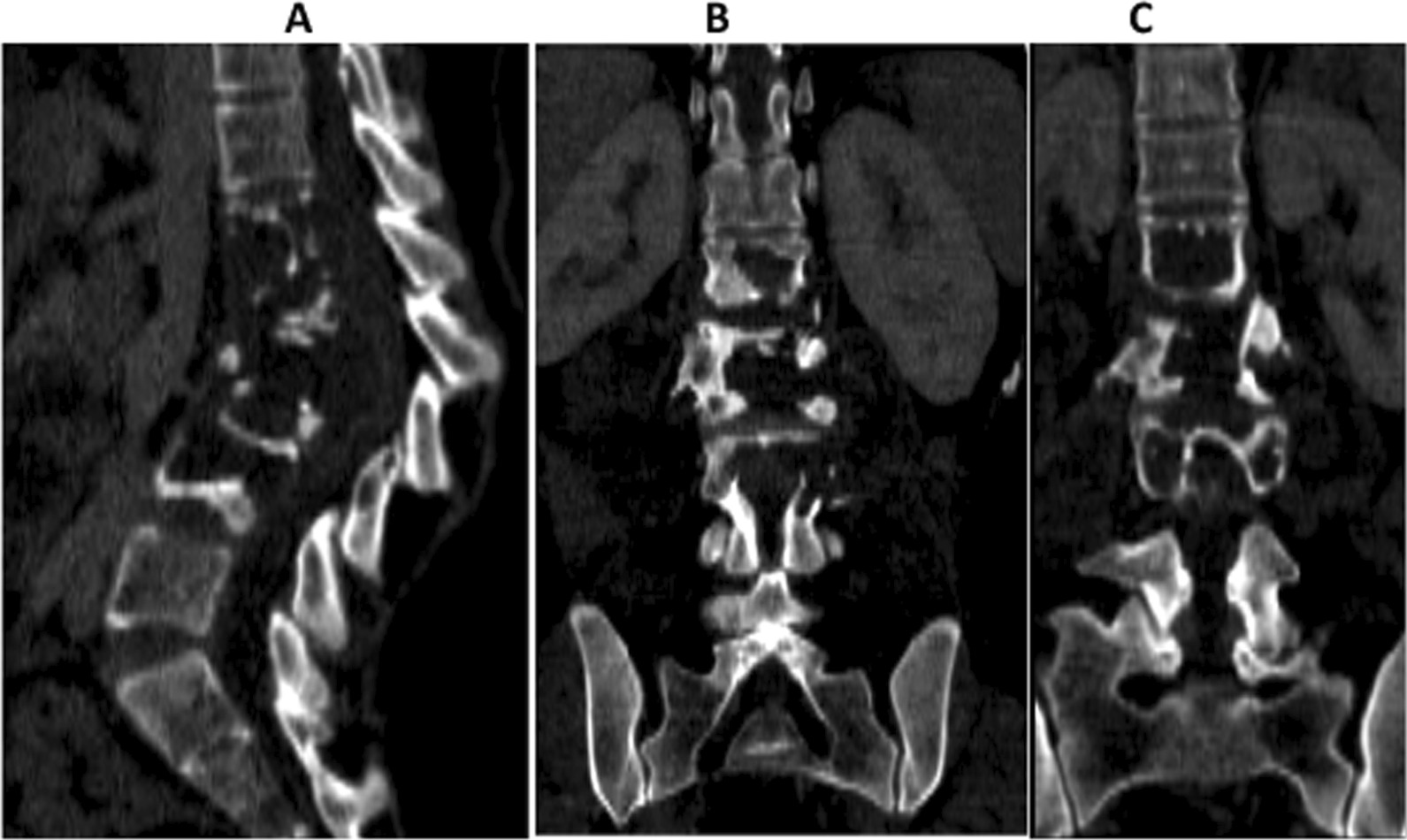
Abdominopelvic CT scan bone window: sagittal (**A**) and coronal (**B** & **C**) plane images show extensive lytic destruction of the L1–L4 vertebral bodies, loss of corresponding intervertebral disc spaces and kyphotic deformity of the affected lumbar spine

**Fig. 3:**
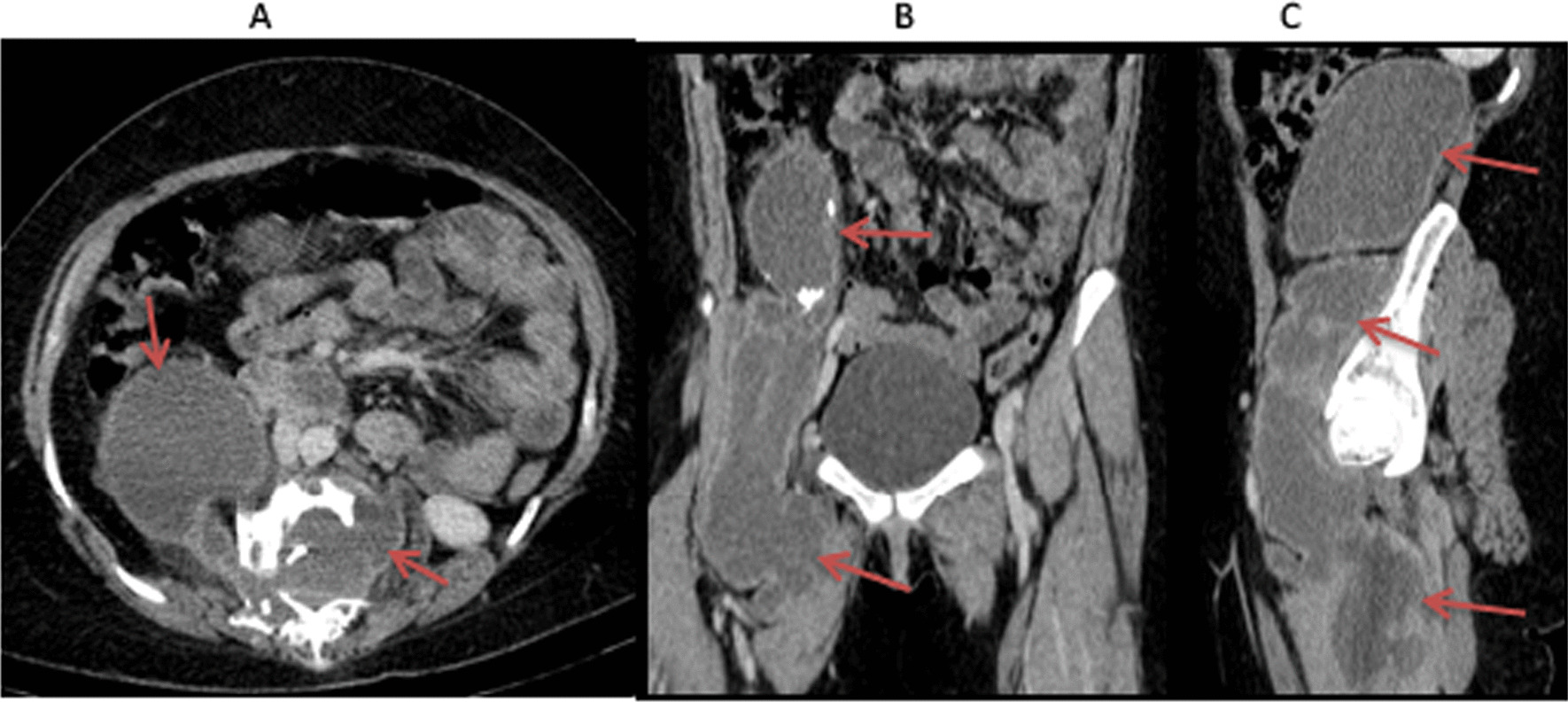
Contrast-enhanced abdominopelvic CT scan: axial (**A**), coronal (**B**) and sagittal (**C**) images demonstrate an extensive paravertebral cystic mass with wall enhancement and calcifications consistent with an abscess (extent of abscess shown by arrows). The abscess extends into the subdural space, and tracks along the retroperitoneal space into the gluteal compartments of the right thigh (best demonstrated on coronal and sagittal images)

An abdominal CT scan done at the regional referral hospital (Figs. 2, 3) showed extensive lytic destruction of L1 to L4 vertebral bodies, with anterior wedging and kyphosis of the involved segment, associated with a large right paravertebral cystic mass that showed calcifications and rim enhancement consistent with a cold abscess. The abscess extended from the third lumbar vertebral level, involving and narrowing the epidural space, tracking down into the retroperitoneal space to the posterior compartment of the right thigh.

## Treatment

The patient was initiated on anti-tuberculosis drugs; 3 tablets of HERZ [isoniazid (75 mg) + ethambutol (275 mg) + rifampicin (150 mg) + pyrazinamide (400 mg)] daily for the intensive phase of 2 months and thereafter continued with isoniazid (75 mg) and rifampicin (150 mg) for a period of 10 months, which she completed on 20 May 2023 (as per Uganda national guidelines on TB treatment).

She also continued with highly active antiretroviral therapy (HAART) [tenofovir (TDF) (300 mg) + Lamivudine (300 mg) and a double dose of dolutegravir (50 mg)] as per the Uganda HIV treatment guidelines. The abscess was successfully drained and she was provided with a lumbar corset for stabilization of the lumbar spine. Spine neurosurgical interventions were not possible due to lack of funds for the operation.

At 2, 6, and 12-month follow-ups, the patient reported improvement and was clinically stable, although she had lower limb weakness and parasthesias that had started during the course of treatment and had not resolved, despite completing anti-TB drugs. Neurosurgical intervention was not yet possible by the time of the last review.

## Discussion

This case represents unusual presentation of Pott disease in a patient with HIV, who had extensive lumbar vertebral destruction and a large cold abscess that had tracked into the retroperitoneal space to the gluteal region, but with no significant neurological deficits at the time of diagnosis. Additionally, it highlights the unique challenges of TB diagnosis in resource-limited settings where access to proper imaging and other diagnostic modalities is limited or not possible, resulting in debilitating effects of late diagnosis in the affected patients. Moreover, those diagnosed may not be able to access all the needed intervention, such as spine surgery, to restore proper neurological function, as was the case here.

Tuberculosis is the leading cause of death among people living with HIV (PLHIV) [6] and the prevalence of extrapulmonary TB, of which Pott disease is a part of, is also highest in this group, owing to the impaired immunological status [7].

Pott disease refers to the destruction of the vertebral body and intervertebral disc by *Mycobacterium tuberculosis* (MTB) and can also be called TB spondylodiscitis [2]. The infection usually spreads from the respiratory tract through the hematogenous system or by lymphatics from paraaortic lymph nodes, with preference for the thoracic and lumbar vertebrae, and will usually involve two or more contiguous vertebral bodies and their respective intervertebral discs [8]. Destruction of the vertebral bodies and intervertebral discs results in vertebral collapse, with kyphosis and gibbus deformity [9]. In our case, all the four lumbar vertebrae and their intervening intervertebral discs were severely destroyed with vertebral collapse.

In extensive disease, the infection spreads to involve the ligaments, paravertebral soft tissue, the epidural space, and formation of a cold abscess. The abscess may compress the spinal cord or nerve roots with resulting neurological deficits, and may extend into the retroperitoneal space [1]. Our patient already had an extensive paravertebral (from L3 level downwards) abscess that was involving the epidural space, but not yet compressing the nerve roots, which is possibly the reason the patient had not yet developed neurological symptoms. In addition, the abscess was extending into the retroperitoneal space and palpable on abdominal examination, tracking down into the posterior compartment of the right thigh. The pressure symptoms from the abscess had caused right lower abdominal pain, which was the main complaint.

The clinical picture is usually non-specific; hence, establishing a diagnosis especially in resource-limited settings where the diagnostic capacity is limited is often difficult [10]. The usual TB symptoms of fever, weight loss, malaise, and night sweats are only present in less than 40% of patients diagnosed with extrapulmonary TB, while back pain and lower limb weakness are the most common symptoms usually present in about 80% and 73% of patients, respectively [1]. If diagnosed late, the patient may already have kyphotic deformity due to collapse of the anterior spinal elements, or even neurological deficits because of the compression of the spinal cord or spinal nerve roots [1]. In our case, the patient had presented with worsening right lower abdominal pain from the expanding retroperitoneal abscess, but seemed unbothered by the back pain, most likely because of the relief from analgesics, antidepressants, and the used herbal medication. The attending health workers in the peripheral facilities had attempted no imaging, such as X-ray, possibly because of unavailability and low index of suspicion. The set back to the case is that we had little control of the patient management, since neurosurgical services are not freely available and subsequent review CT scans also depend on whether the patient can pay for them, since it is not a free service.

## Conclusion

Unusual presentation of Pott disease, coupled with limited access to basic diagnostic equipment like X-rays, poses a major challenge in resource-limited settings due to missed or late diagnoses of the disease.

Therefore, there is need for deliberate effort to routinely hold continuous medical education (CME) activities for health workers, and equip health facilities with basic diagnostic equipment to facilitate diagnosis of extrapulmonary TB with unusual presentation.

## Data Availability

The data is available and accessible.

## Abbreviations

TB: Tuberculosis
HIV: Human Immunodeficiency Virus
ART: Antiretroviral therapy
MRRH: Mbarara Regional Referral Hospital
TLE: Tenofovir + lamivudine + efavirenz
TLD: Tenofovir + lamivudine + dolutegravir
VL: Viral load
mmHg: Millimeters of mercury
CT: Computed tomography
ZN: Ziehl–Neelsen
MTB: Mycobacterium tuberculosis
CBC: Complete blood count
